# Supplementary material for: Migrasome-Related Genes as Potential Prognosis and Immunotherapy Response Predictors for Colorectal Cancer
Source: Biomedicines. 2025 Mar 26;13(4):799. doi: 10.3390/biomedicines13040799 (PMC12024535; doi:10.3390/biomedicines13040799)
Supplement: Supplementary file 1 [file biomedicines-13-00799-s001.zip › biomedicines-3472083-supplementary.pdf]

Supplementary Figure Legends:

Supplementary Fig. S1 Identification of migrasome-related module genes. (a) Hierarchical clustering of samples based on gene expression profiles. (b) Selection of the optimal soft threshold for network construction. Left, a scale-free fit index of 0.85 is achieved, meeting the minimum requirement for constructing a scale-free network. Then a soft threshold of 26 is chosen for subsequent analysis. Right, network connectivity under different soft threshold values. (c) Identification of co-expression modules. Upper, hierarchical clustering dendrogram of genes. Lower, identified gene co-expression modules (network modules). The clustering dendrogram groups genes based on their expression similarity, and closely related genes (clustered on the same branch) are assigned to the same module, as indicated by the corresponding colors below.

Supplementary Fig. S2 Function enrichment analysis of the 6 migrasome-related candidate genes. (a) Identification of highly variable genes. (b) PCA for dimensionality reduction. (c) Dimension selection using the JackStraw method. (d) Boxplot of migrasome-associated ssGSEA scores between high-score and low-score cell groups.

Supplementary Fig. S3 Survival analysis of the risk model. (a) LASSO regression analysis. Left, relationship between the number of feature genes and the proportion of explained deviance (residual variance). Right, cross-validation error against log (Lambda). Lambda is the regularization parameter in LASSO regression. (b) Risk curve (left) and scatter plot (right) of high- and low-risk groups in the training set. (c) Risk curve (left) and scatter plot (right) of high- and low-risk groups in GSE17537 dataset.

Supplementary Fig. S4 Prognostic performance analysis of the risk model. (a) Calibration curves for nomogram-predicted 1-, 2-, and 3-year survival probabilities. (b) Decision Curve Analysis (DCA) for the Nomogram model at 1-, 2-, and 3-year time points.

Supplementary Fig. S5 Immunotherapy response prediction of the risk model. (a) Immune, stromal, and ESTIMATE scores of high- and low-risk groups in the TCGA-CRC dataset. (b) Immune infiltration analysis of 28 immune cells in high- and low-risk groups in the TCGA-CRC dataset.

Supplementary Fig. S6 Stratified survival curves of high- and low-expression groups. (a) Survival curves of high- and low- CD274, LAG3, LGALS9 and PDCD1 expression of the CRC patient.

Supplementary Figure S1

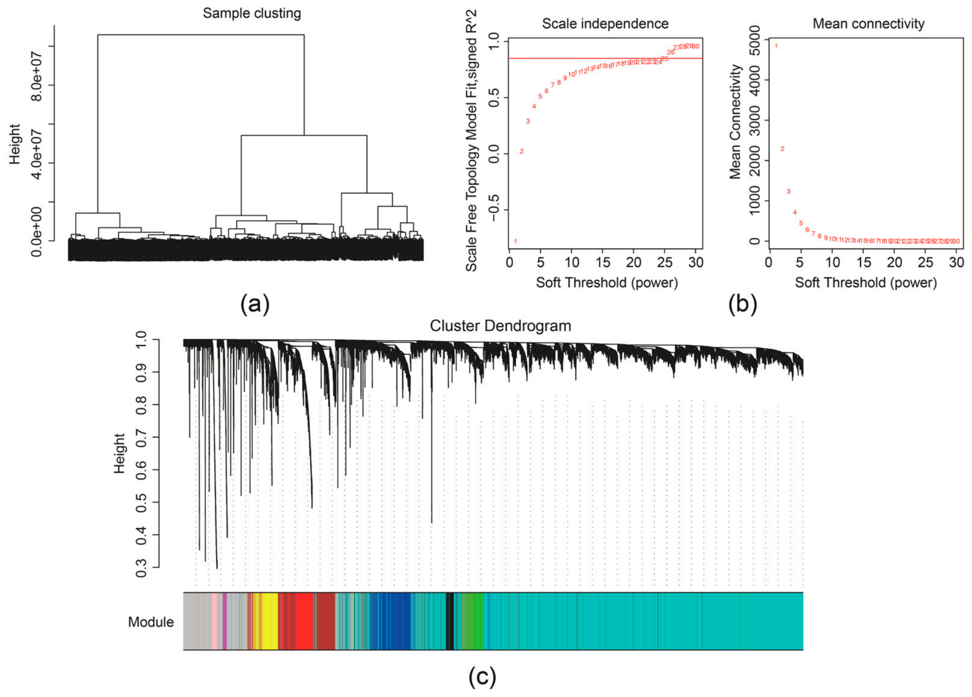

Supplementary Figure S2

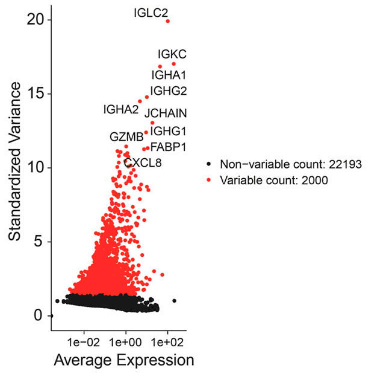

(a)

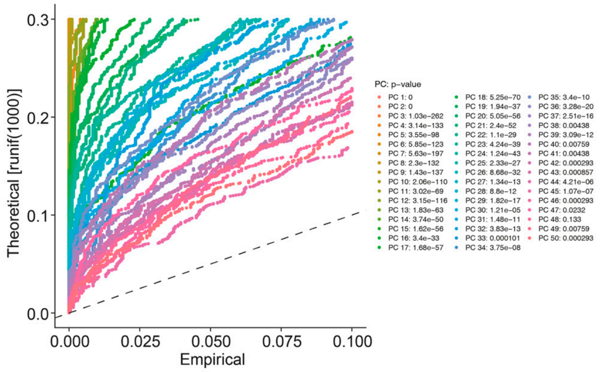

(b)

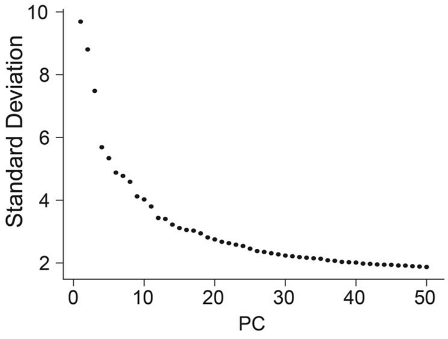

(c)

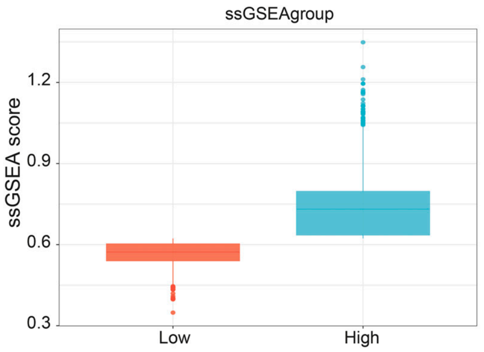

(d)

Supplementary Figure S3

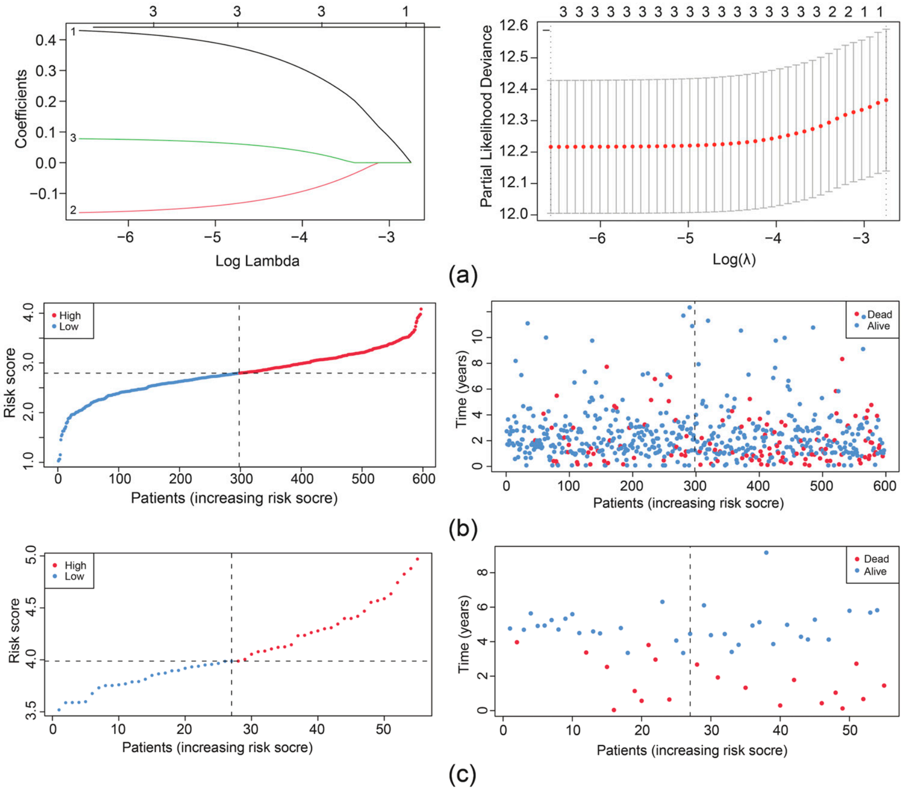

Supplementary Figure S4

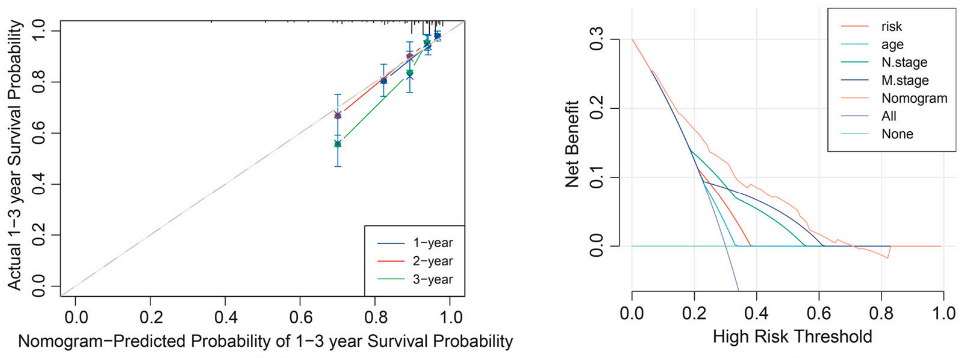

(a) (b)

Supplementary Figure S5

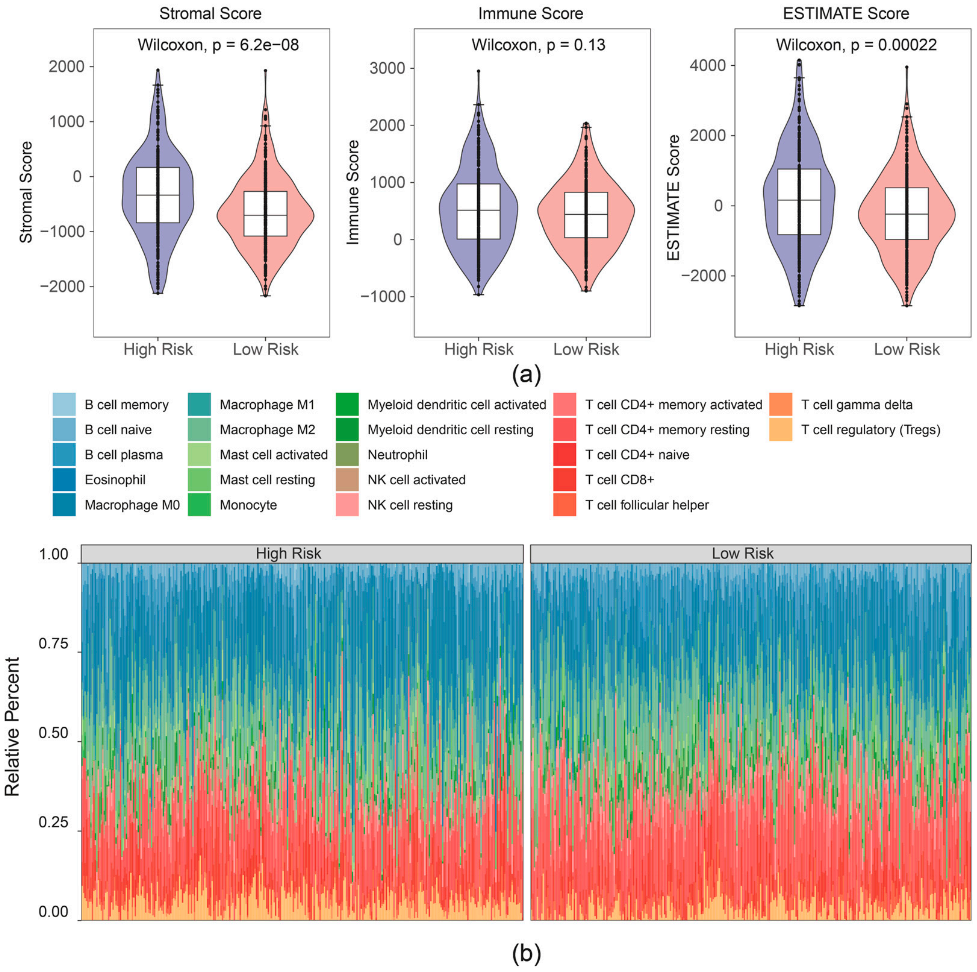

Supplementary Figure S6

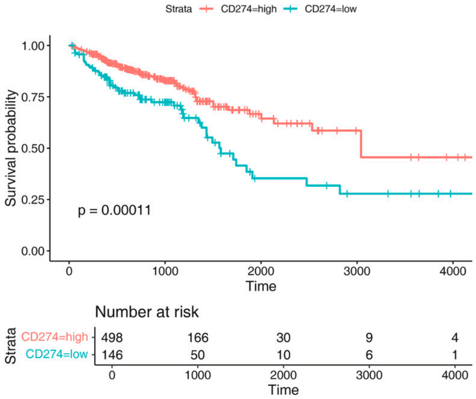

(a)

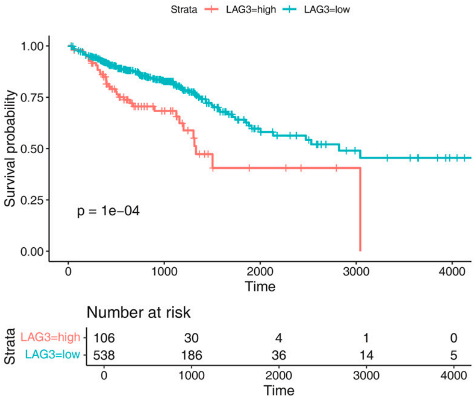

(b)

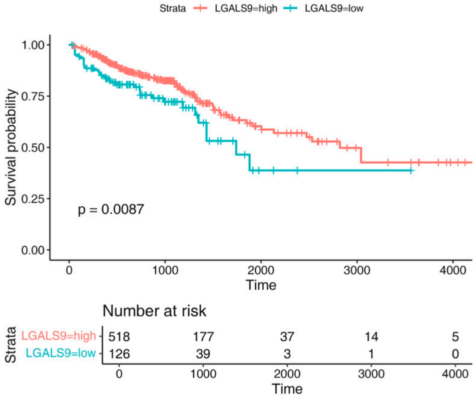

(c)

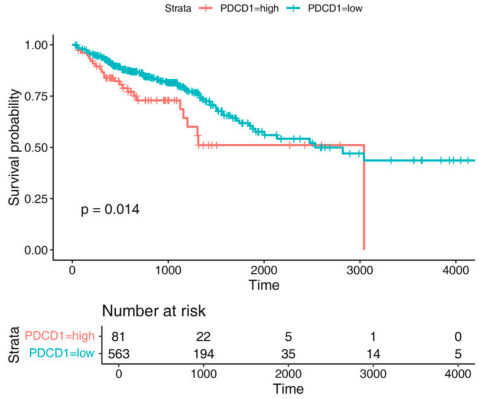

(d)

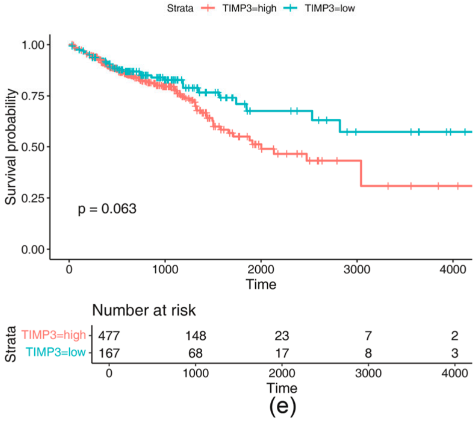

(e)
